# Insights into amyloid precursor protein target through PPI network analysis

**DOI:** 10.6026/973206300200140

**Published:** 2024-02-29

**Authors:** Annu Grewal, Deepak Sheokand, Raveena Chauhan, Vandana Saini, Ajit Kumar

**Affiliations:** 1 Toxicology and Computational Biology Group, Centre for Bioinformatics, Maharshi Dayanand University, Rohtak, Haryana, India, 124001

**Keywords:** Protein-protein interaction, beta-amyloid (Aβ) accumulation, Cytoscape, STRING

## Abstract

Alzheimer's disease (AD) is the leading cause of dementia worldwide with therapeutic lacunae till date. The beta-amyloid (Aβ) accumulation triggers AD pathogenesis, though clinical trials lowering Aβ have not altered disease outcomes suggesting other interacting factors to be identified for drug design of AD. Therefore, it is of interest to identify potential hub proteins interlinked with disease-driving pathways using a network-based approach for AD therapeutic designing. Literature mining was done to identify proteins implicated in AD etiology. Protein-protein interactions (PPIs) were retrieved from the STRING database and merged into a single network using Cytoscape 3.10.1. The hub proteins involved in AD etiology were predicted based on the topological algorithms of CytoHubba. Six major proteins, with STRING database identifiers - APP, BACE1, PSEN1, MAPT, APOE4 and TREM2, were identified to be involved in AD pathogenesis. The merged network of PPIs of these proteins contained 51 nodes and 211 edges, as predicted by Analyzer module of Cytoscape. The Amyloid precursor protein (APP) emerged as the highest-scoring hub protein across multiple centrality measures and topological algorithms. Thus, current data provides evidence to support the ongoing investigation of APP's multifaceted functions and therapeutic potential for AD.

## Background:

Alzheimer's disease (AD) is the leading cause of neurodegenerative disorders and is responsible for 60-70 percent of dementia cases worldwide. [1]. Recent evidence across systemic evaluations underscores the immensity of the AD crisis confronting global health and healthcare systems in the 21st century. Current models estimate nearly 75 million people worldwide suffered from some AD-related neurocognitive disability in 2023, with projections of 139 million cases by 2050 if therapeutic gaps continue [2]. Global AD data demands urgent attention toward developing generalizable and cost-effective medications for AD prevention and treatment. The drugs available for AD treatment, including cholinesterase inhibitors (Donepezil, Rivastigmine, Galantamine etc.) and antagonists of the N-methyl-D-aspartate receptor (Memantine), can only improve cognition for a limited period but cannot stop or reverse the disease progression [3]. While current medications provide modest and symptomatic relief for some Alzheimer's patients in the early to middle stages, they come with limitations. Cholinesterase inhibitors in particular can cause gastrointestinal side effects like nausea, vomiting, and diarrhea. Other side effects like headaches, insomnia, and dizziness may also occur [4]. Development of drugs to slow or stop the neurodegeneration and progression of Alzheimer's remains a key priority. Combination therapies targeting multiple aspects of disease or identifying the main protein, interlinked to all the disease-driving pathways, hold promise for the future.

Multiple equivocal hypotheses (Amyloid cascade hypothesis, Tau hypothesis, mitochondrial dysfunction hypothesis and Neuroinflammation hypothesis) have been proposed to explain the underlying mechanisms of memory loss and cognitive decline in the pathogenesis of AD [4]. The amyloid cascade hypothesis proposes that the accumulation of beta-amyloid (Aβ) peptides due to impaired clearance triggers a cascade leading to AD pathology and symptoms. Aβ peptides result from the proteolytic cleavage of the amyloid precursor protein (APP) by various secretases and the peptide Aβ42 is more prone to aggregation into plaques [5,6]. However, the hypothesis has limitations; clinical trials targeting Aβ have not successfully treated AD, suggesting other factors are likely involved [7]. The tau hypothesis postulates that the buildup of abnormal tau proteins in the brain is the primary causal factor in the development of AD, rather than Aβ [8]. The elevated number of neurofibrillary tangles (NFTs) is even detected in some pre-amyloid cases of early Alzheimer's, termed primary aging-related tauopathy [9]. This suggests tau pathology can precede Aβ. However, tau protein accumulation also occurs in other neurodegenerative diseases [10]. Therefore, tau pathology alone is not specific to AD, and underlying mechanisms may differ across diseases. Mitochondrial dysfunction has been linked to the accumulation of Alzheimer's hallmarks like Aβ plaques and NFTs [11]. However, it remains unclear whether mitochondrial dysfunction is a cause or a consequence of AD pathogenesis [12]. Preclinical studies in mouse and rat models indicate AD progression can be slowed by targeting mitochondria and restoring function through antioxidants [13]. Targeting mitochondria and oxidative stress shows promise for slowing AD, but the intricacies are not yet fully characterized. Neuroinflammation is known to play a significant role in AD pathogenesis [14]. Brains affected by AD exhibit heightened levels of inflammatory markers such as cytokines, pointing to the existence of persistent minor brain inflammation [15]. Some research indicates that inflammation starts early and adds to the progression of pathological changes, while other studies propose that inflammation is a subsequent immunological reaction to nerve cell damage that has already occurred [16]. Anti-inflammatory drugs like non-steroidal anti-inflammatory drugs (NSAIDs) and statins have been tested for AD treatment with mixed results [17]. This suggests inflammation is likely not the sole driver of AD. More research is needed to unravel the timing and interrelation between the hypotheses related to AD pathology. Therefore, it is if interest to identify the hub protein(s) responsible for AD pathogenesis, using literature mining and network-based approaches.

## Materials and Methodology:

## Literature mining:

The KEGG pathway database [18] was mined for the identification of the main protein targets responsible for the pathological state of AD (map: hsa05010). The proteins identified to be involved in different mechanisms of AD progression were selected for further studies of their interrelated interactions.

## Protein-protein interactions:

The protein-protein interaction (PPI) networks of the identified proteins, involved in AD etiology, were retrieved using the STRING database. The STRING database is based on known interactions retrieved from experimental and curated databases; predicted interactions derived using gene fusions, neighbourhood and co-occurrence criteria, and other interactions retrieved from text mining, protein homology and co-expression [19]. The retrieved PPI networks of all the proteins were analysed for the number of nodes (representing query proteins), number of edges (representing protein-protein associations), average node degree, expected number of edges, average local clustering coefficient and PPI enrichment p-value, using Analysis module of STRING database.

## Network generation and analysis:

The PPI networks of the identified proteins, involved in AD pathology and as retrieved from the STRING database, were merged into a single PPI network map at a confidence score of 0.40, using Cytoscape 3.10.1 [20]. The Analyzer tool of Cytoscape 3.10.1 was used to analyse the merged network. The Analyzer predicted the summary of the merged network and provided statistics of the number of edges, nodes, average number of neighbours, clustering coefficient, network heterogeneity, network centralisation, characteristics path length etc. The single merged network was used for further studies of hub protein identification.

## Hub-protein identification:

The single merged network generated by merging the PPI networks of the identified proteins was studied for its topology using local and global algorithms of the CytoHubba module [21]. The 4 local rank methods of the CytoHubba i.e. Degree, Maximal clique centrality (MCC), Maximum neighborhood component (MNC), and Density of maximum neighborhood component (DMNC) only consider the relationship between the node and its direct neighbors, therefore the global rank methods involving Edge percolated component (EPC) and 6 centralities i.e. Bottleneck, EcCentricity, Closeness, Radiality, Betweenness and Stress which examine the relationship between the node and the entire network, were also used for the hub-protein identification. The merged network was selected as the target network and the nodes' score was calculated for the top 10 nodes of Hubba. The nodes' score was analyzed for all the topological algorithms of CytoHubba, and hub protein was identified based on the average scoring of all the algorithms.

## Results and Discussion:

## Literature mining:

A total of 6 proteins with STRING database identifiers - APP, BACE1, PSEN1, MAPT, APOE4, and TREM2, were identified to be involved in different hypothetical mechanisms of AD progression using the KEGG pathway database. APP (amyloid-precursor protein) is the initial protein of amyloid-cascade hypothesis and its processing in the amyloidogenic pathway is mediated by BACE1 (β-secretase) and PSEN1 (Presenilin-1). Furthermore, BACE1 and PSEN1 were found to be associated with the neuroinflammatory [22] and Tau [23] hypotheses, respectively. MAPT (Microtubule-associated protein tau) is responsible for the tau hypothesis and formation of NFTs. APOE4 (Apolipoprotein E4) is found to be involved in various mechanisms like neuroinflammation, tau pathology and decreased Aβ clearance [24]. TREM2 (Triggering receptor expressed on myeloid cells 2) is a microglial transmembrane receptor associated with the neuroinflammatory hypothesis of AD [25].

## Protein-protein interactions:

**Figure 1:**
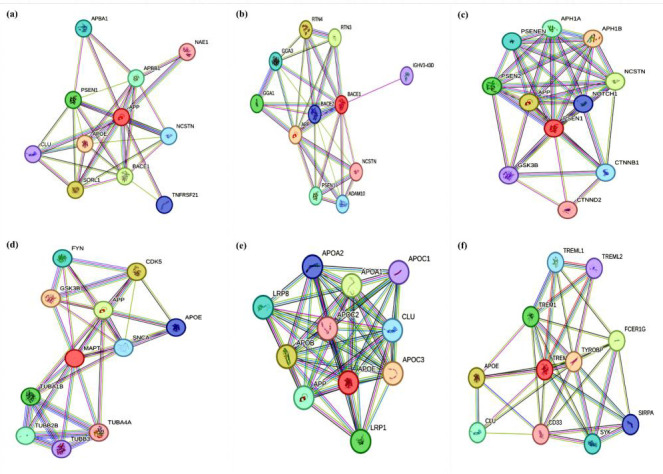
Protein-protein interactions of the identified proteins a) APP; b) BACE1; c) PSEN1; d) MAPT; e) APOE4; f) TREM2 (Colored nodes represent query proteins and first shell of interactions, edges represent various known and predicted interactions determined using gene neighborhood, gene fusions and gene co-occurrence).

The STRING database provided functional and binary associations for each protein identified using literature mining of the KEGG database (APP, BACE1, PSEN1, MAPT, APOE4, and TREM2), in the form of PPI networks. The PPIs were analysed for the known and predicted interactions and the PPIs above an average clustering coefficient of 0.8 were selected for further studies of hub-protein identification (Figure 1). An average of 11 nodes (proteins involved in the network) were obtained for each PPI network and the number of edges (associations determined from databases, gene neighbourhood, gene fusions, gene co-occurrence, co-expression, and protein homology) varied from 32 to 52, more than the expected number of edges (Table 1). The average node degree was found to vary from 5.82 to 9.45 and the PPI enrichment p-value was observed to be very small for each network suggesting functional relation of the proteins involved in a network. APP was observed to have maximum associations with NCSTN (Nicastrin), BACE1 and PSEN1, hence directing towards the amyloidogenic processing as the main pathway (Figure 1a). Similarly, the PPI network of BACE1 was also observed for maximum interactions with APP (Figure 1b). The PPI network of PSEN1 showed maximum interactions of PSEN1 with PSEN2 and PSENEN, pointing towards Notch and Wnt signaling cascades (Figure 1c). The PPI network of MAPT showed maximum interactions of MAPT with CDK5 and tubulin subunits, targeting neuronal health and microtubule associations (Figure 1d). APOE had maximum associations with APP, LRP1 and APOB mainly targeting APP processing and endocytosis (Figure 1e). TREM2 had maximum associations with TYROBP, TREML1 and TREML2 which are involved in tyrosine kinase signalling mediating cell activation and immunological processing (Figure 1f).

## Network generation and analysis:

**Figure 2:**
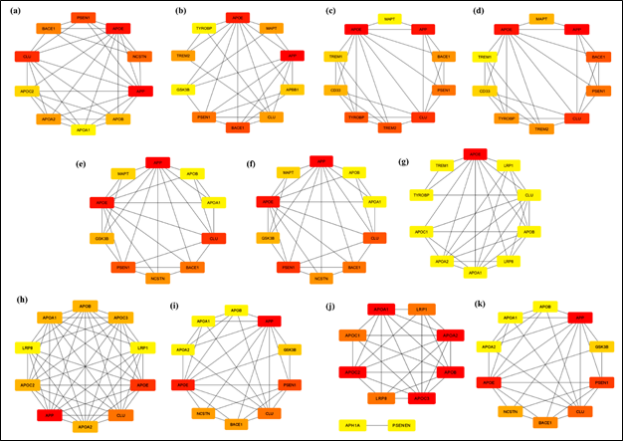
Top 10 hub proteins identified using a) MCC; b) MNC; c) DMNC; e) Degree; f) EPC; g) Bottleneck; h) Stress; i) Betweenness; j) Radiality; k) Closeness; l) Eccentricity topological algorithms (The node scores are varying from red to orange and yellow colors, red colored node being the highest scoring node and yellow colored node being the lowest scoring node)

The PPIs determined from the STRING database were merged into a single network using Cytoscape 3.10.1 and analyzed using the Analyzer of Cytoscape 3.10.1. The merged network was obtained with 51 nodes, 211 edges, an 8.275 average number of neighbours, a characteristic path length of 2.228 and a clustering coefficient of 0.797. The merged network was further used for CytoHubba nodes' score calculation and top 10 nodes were predicted using local (Table 2) and global topological algorithms (Table 3). Firstly, scores from all 11 methods (MCC, MNC, DMNC, Degree, EPC, Bottleneck, EcCentricity, Closeness, Radiality, Betweenness and Stress) were generated and the top-ranked nodes of each method were predicted in a graphical form (Figure 2a-k). Methods like EPC (Figure 2a), Stress (Figure 2c), Betweenness (Figure 2d), Radiality (Figure 2e) and MCC (Figure 2h) assigned higher scores to highly interactive, high-degree proteins and lower scores to low-degree proteins with few interactions. Similar outputs were observed for these 5 algorithms as APP, APOE and CLU were observed to be in the top 3 nodes having the highest scores as the most essential proteins. However, methods like Eccentricity (Figure 2g) and DMNC (Figure 2j) identified low-degree essential proteins, and similar association scores of nodes were obtained from both the methods.

## Hub-protein identification:

Three of the local topological algorithms (MCC, MNC and Degree) predicted APP as the top-scoring node, whereas DMNC provided APOA1 as the top-scoring node. Similarly, the global topological algorithm EPC and four out of six centralities (Betweenness, Radiality, Closeness and Bottleneck) predicted APP as the top-scoring node, Stress predicted APP as the second-highest scoring node, whereas EcCentricity predicted APOE as the top-scoring node and all other nodes were having the same score. Based on the average scores of all the topological algorithms, APP was predicted as the hub-protein (Table 2 and Table 3,Figure 2). The present network findings reinforce APP's extensive connectivity to known AD risk proteins, supporting ongoing research into its multifaceted functions and contributions to neurodegeneration.

## Conclusion:

We used literature mining and network-based approach to identify amyloid precursor protein (APP) as a potential hub protein and a key contributor to AD pathogenesis. Of the proteins analyzed, APP emerged as having the most interactions and a central role within the merged protein-protein interaction network. Data shows APP's extensive connectivity to known AD risk proteins, supporting ongoing investigation into its multifaceted functions and contributions to neurodegeneration. Data further provide a good platform but require careful interpretations, during translation studies for AD therapeutics, given the complexity of AD etiology and should integrate multi-omics data sources to elucidate the interrelations between protein pathways underlying the amyloid, tau, mitochondrial dysfunction, and neuroinflammation hypotheses implicated in disease progression.

## Figures and Tables

**Table 1:** Protein-protein interaction network parameters for the selected proteins

**Network parameters**	**Score for APP**	**Score for BACE1**	**Score for PSEN1**	**Score for MAPT**	**Score for APOE4**	**Score for TREM2**
Number of nodes	11	11	11	11	11	11
Number of edges	33	32	40	33	52	36
Average node degree	6	5.82	7.27	6	9.45	6.55
Avg. local clustering coefficient	0.857	0.871	0.855	0.841	0.952	0.826
Expected number of edges	12	11	15	16	13	12
PPI enrichment p-value	5.37E-07	2.15E-07	3.59E-08	0.000124	2.22E-16	2.11E-08

**Table 2:** CytoHubba nodes' score of the top 10 nodes obtained from global topological algorithms

**Ranks**	**EPC protein**	**EPC score**	**Bottleneck protein**	**Bottleneck score**	**Stress protein**	**Stress score**	**Betweenness protein**	**Betweenness score**	**Radility protein**	**Radiality score**	**Closeness protein**	**Closeness score**	**Eccentricity protein**	**Eccentricity score**
1	APP	20.27	APP	43	APOE	3552	APP	1066.2	APP	3.74	APP	42.6	APOE	0.5
2	APOE	19.3	APOE	10	APP	2910	APOE	633.66	APOE	3.56	APOE	36.5	APOA1	0.33
3	CLU	17.6	BACE1	8	CLU	1622	CLU	226.26	CLU	3.38	PSEN1	32.8	LRP1	0.33
4	PSEN1	17.07	PSEN1	6	TYROBP	1288	BACE1	197.26	PSEN1	3.32	CLU	32.6	APOB	0.33
5	NCSTN	15.8	CLU	5	TREM2	1288	PSEN1	169.39	BACE1	3.26	BACE1	31.3	CLU	0.33
6	BACE1	15.5	TREM2	5	PSEN1	1032	TYROBP	143.5	NCSTN	3.24	NCSTN	30.8	LRP8	0.33
7	APOA2	15.3	MAPT	5	BACE1	870	TREM2	143.5	GSK3B	3.2	GSK3B	29.5	TREM1	0.33
8	APOB	15.24	APBB1	3	CD33	766	MAPT	134.9	MAPT	3.18	MAPT	29	APOA2	0.33
9	APOC2	15.07	TYROBP	2	TREM1	634	CD33	75.14	APOA1	3.12	APOA	28.5	APOC1	0.33
10	APOA1	14.8	GSK3B	2	MAPT	458	TREM1	72.23	APOB	3.12	APOB	28.5	TYROBP	0.33

**Table 3:** CytoHubba nodes' score of the top 10 nodes obtained from local topological algorithms

**Ranks**	**MCC protein**	**MCC score**	**MNC protein**	**MNC score**	**DMNC protein**	**DMNC score**	**Degree protein**	**Degree score**
1	APP	127444	APP	37	APOA1	0.83	APP	37
2	APOE	121974	APOE	23	APOB	0.83	APOE	23
3	CLU	121104	PSEN1	18	APOA2	0.83	PSEN1	18
4	APOA1	120960	CLU	16	APOC2	0.83	CLU	16
5	APOB	120960	NCSTN	14	APOC3	0.83	BACE1	15
6	APOA2	120960	BACE1	14	LRP1	0.81	NCSTN	14
7	APOC2	120960	GSK3B	11	LRP8	0.81	GSK3B	11
8	APOC3	120960	APOA1	10	APOC1	0.81	APOA1	10
9	LRP1	40320	APOB	10	APH1A	0.76	APOB	10
10	LRP8	40320	APOA2	10	PSENEN	0.76	APOA2	10

## References

[1] Leng F, Edison P (2021). Nat Rev Neurol..

[2] Gustavsson A (2023). Alzheimer's Dement..

[3] Huang L (2020). J Biomed Sci..

[4] Nasb M (2024). Aging Dis..

[5] Selkoe DJ (2001). J Alzheimers Dis..

[6] Selkoe DJ, Hardy J (2016). EMBO Mol Med..

[7] Cummings J (2016). Alzheimers Res Ther..

[8] Guo T (2017). Acta Neuropathol..

[9] Crary J.F (2014). Acta Neuropathol..

[10] Martínez-Maldonado A (2021). J Alzheimers Dis..

[11] Fang E (2019). Nat Neurosci..

[12] Burtscher J (2022). Prog Neurobiol..

[13] Yang P (2020). Biomaterials..

[14] Kinney J.W (2018). Alzheimers Dement (N Y)..

[15] Zotova E (2010). Alzheimers Res Ther..

[16] Ransohoff RM (2016). Science..

[17] Aisen P.S (2003). JAMA..

[18] Kanehisa M (2010). Nucleic Acids Res..

[19] Szklarczyk D (2023). Nucleic Acids Res..

[20] Shannon P (2003). Genome Res..

[21] Chin C.H (2014). BMC Syst Biol..

[22] Koelsch G (2017). Molecules..

[23] Yang Y (2023). Int J Mol Sci..

[24] Hunsberger H.C (2019). Neuronal Signal..

[25] Kulkarni B (2021). Mol Neurobiol..

